# Transmission Stages Dominate Trypanosome Within-Host Dynamics during Chronic Infections

**DOI:** 10.1016/j.chom.2011.03.013

**Published:** 2011-04-21

**Authors:** Paula MacGregor, Nicholas J. Savill, Deborah Hall, Keith R. Matthews

**Affiliations:** 1 Centre for Immunity, Infection, and Evolution, Institute for Immunology and Infection Research, School of Biological Sciences, University of Edinburgh, King's Buildings, West Mains Road, Edinburgh EH9 3JT, UK

## Abstract

Sleeping sickness is characterized by waves of the extracellular parasite *Trypanosoma brucei* in host blood, with infections continuing for months or years until inevitable host death. These waves reflect the dynamic conflict between the outgrowth of a succession of parasite antigenic variants and their control by the host immune system. Although a contributor to these dynamics is the density-dependent differentiation from proliferative “slender forms” to transmissible “stumpy forms,” an absence of markers discriminating stumpy forms has prevented accurate parameterization of this component. Here, we exploit the stumpy-specific PAD1 marker, which functionally defines transmission competence, to quantitatively monitor stumpy formation during chronic infections. This allows reconstruction of the temporal events early in infection. Mathematical modeling of these data describes the parameters controlling trypanosome within-host dynamics and provides strong support for a quorum-sensing-like mechanism. Our data reveal the dominance of transmission stages throughout infection, a consequence being austere use of the parasite's antigen repertoire.

## Graphical abstract

**Graphical Abstract:**

**Figure:**
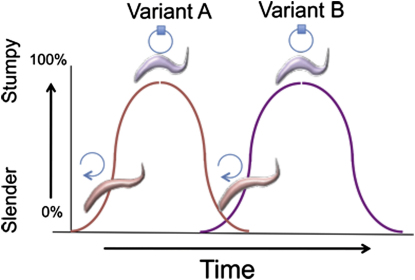

**Highlights:**

► Quantification of trypanosome transmission stages in chronic infections ► The temporal order of developmental events early in infection defined ► Mathematical data modeling provides support for parasite quorum sensing ► The dominance of transmission stages has key implications for VSG switch frequency

## Introduction

The success of a vector-borne parasite depends upon its ability to survive in its mammalian host and to ensure transmission to future hosts. Although these ambitions might be complementary, they can also be in conflict because parasite growth can damage the host and so limit transmission potential (Frank, 1996). An excellent example of this conflict is the sleeping sickness parasite, *Trypanosoma brucei*, which is sustained in the mammalian bloodstream by a sophisticated antigenic variation system until transmission by tsetse flies. Specifically, African trypanosomes express a repertoire of variant surface glycoprotein (VSG) coats, encoded by VSG genes which are expressed individually from about 20 telomeric expression sites (ES), of which only one is active at a time. Antigen switching requires either a change in ES activity or recombination of one of several thousand silent VSG gene sequences into an active ES. Recombination involves either translocation of an intact gene mediated by homologous flanking sequences or the assembly of functional mosaic genes (Taylor and Rudenko, 2006). The different probabilities of gene activation by these routes generate a hierarchy of antigen-type expression during infections, contributing to the progressive waves of parasitemia characteristic of trypanosome infection (Gjini et al., 2010; Lythgoe et al., 2007).

Each wave of parasitemia also comprises the differentiation from slender to stumpy forms (Matthews, 2005). Slender forms are proliferative and rapidly establish the parasitemia, whereas stumpy forms are proposed to arise via a density-sensing mechanism involving an unidentified parasite-derived soluble signal, stumpy induction factor (Vassella et al., 1997). Stumpy forms are irreversibly committed to cell-cycle arrest in the bloodstream, thereby limiting parasite numbers and prolonging host survival. Unlike slender forms, stumpy forms are also competent for transmission by tsetse flies, the vector for sleeping sickness. This is because they can survive the stresses of tsetse uptake (Nolan et al., 2000; Robertson, 1912; Turner et al., 1988) and are uniquely sensitive to the associated environmental cues. These include the metabolites citrate, or *cis*-aconitate, which stumpy forms perceive when at reduced ambient temperature (Engstler and Boshart, 2004) through their surface expression of PAD proteins, a family of carboxylate transporters (Dean et al., 2009). Of these, PAD1 is stumpy specific, providing a marker functionally relevant to transmission competence able to distinguish differentiated parasites. This allows the quantitative discrimination of transmissible stumpy forms, analyses previously limited by the necessity for empirical and subjective observation of cell-type morphology. Here, we have accurately quantitated differentiated parasites in chronic infections, enabling the key parameters governing trypanosome infection dynamics to be dissected objectively. Our results demonstrate the dominance of transmission stages during long-term infection, predicting that trypanosomes reduce their VSG switch frequency in established infections by restricting the number of proliferative slender forms. Elegantly, this has the potential to optimize parasite transmission probability while prolonging the survival of the host and parasite population.

## Results

### Quantitative Analysis of Trypanosome Chronic Infections

**Figure 1:**
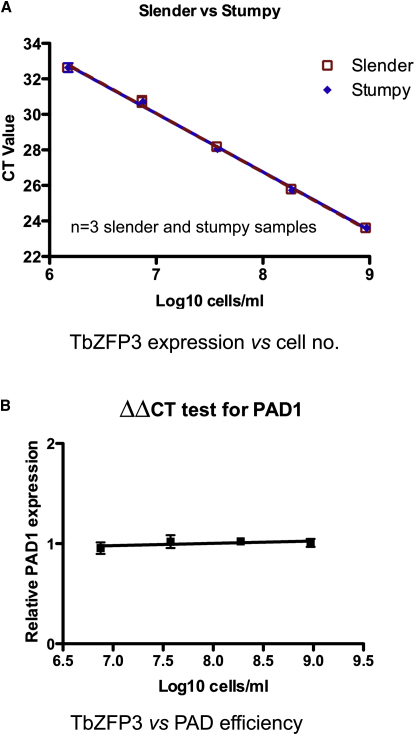
Validation of qRT-PCR Method (A) Validation of the constitutive expression of *TbZFP3* mRNA. *TbZFP3* specific primers were used to amplify cDNA derived from serial dilutions of samples that were derived from populations that were either slender or stumpy in morphology (these having been derived from mouse infections). Triplicate assays are shown, which demonstrated that neither the slope nor the intercept of the regression lines of *TbZFP3* expression in the slender and stumpy samples were significantly different (F_1,25_ = 0.048, p = 0.8276; and F_1,26_ = 0.004, p = 0.9493) over a 1000-fold range (1.5 × 10^6^ to 9 × 10^8^ trypanosomes/ml), with a combined PCR efficiency of 100.92%. This established that the level of mRNA for *TbZFP3* was equivalent in each cell type. (B) The efficiency of amplification of amplicons detecting either *TbZFP3* or *PAD1* was compared over serial dilutions of stumpy forms (7.3 × 10^6^ to 9 × 10^8^ trypanosomes/ml). Equivalent amplification efficiency was observed for each target transcript, there being a semi-log regression line of slope 0.023. Error bars representing standard error of the mean (SEM) are included for the triplicate assays, these being difficult to visualize on the derived plots due to the high level of reproducibility of the assays.

To analyze trypanosome within-host dynamics, we established infections by infecting mice with 1000 AnTat1.1 trypanosomes from a donor infection and monitored them daily by microscopy to estimate parasite number. RNA was extracted to measure the expression of *PAD1* and the transcript for the constitutively expressed RNA-binding protein, *TbZFP3* (Paterou et al., 2006), enabling accurate scoring of cell-type differentiation and cell number, respectively. Although complicated by sequence similarity among members of the differentially expressed *PAD* gene family, a region spanning from the *PAD1* open reading frame to its 3′ untranslated region (UTR) demonstrated sufficient discrimination to accurately profile *PAD1*-specific expression by qRT-PCR. By the same approach, the equivalent expression of *TbZFP3* messenger RNA (mRNA) between slender and stumpy forms was confirmed over a 1000-fold range (1.5 × 10^6^ to 9 × 10^8^ trypanosomes/ml) (Figure 1A). The amplification efficiencies for the *TbZFP3* and *PAD1* transcripts were also shown to be suitably similar over a range of 7.3 × 10^6^ to 9 × 10^8^ trypanosomes/ml for the use of the ΔΔCT method to calculate relative *PAD1* expression per cell (Figure 1B).

**Figure 2:**
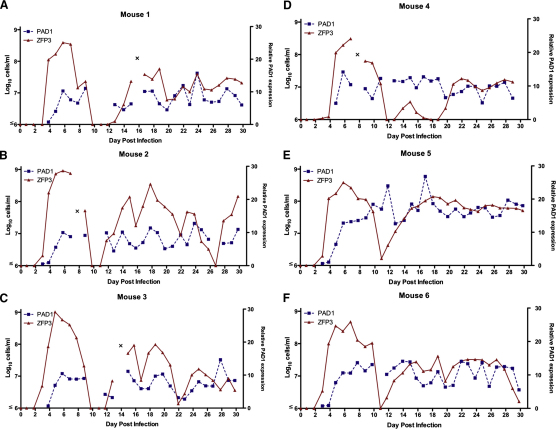
Dynamics of Infections with *Trypanosoma brucei* in Mice Parasite number (derived by qRT-PCR analysis of the constitutive transcript, *TbZFP3*) (left-hand axis) and relative *PAD1* expression per cell (relative to mouse 5, day 4 as a common standard) (right-hand axis) for each mouse (A–F) on each day of infection. Data were not derived prior to day 3 due to the parasite numbers being below the detectable threshold. See also Figure S1.

**Figure 3:**
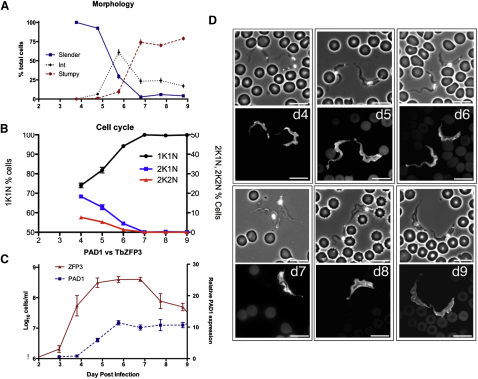
Analysis of the First Wave of Parasitemia in Six Mice from Day 4 to Day 9 after Infection (A) Microscopical analysis of trypanosome morphologies. (B) Proportion of distinct cell-cycle types (K, kinetoplast; N, nucleus). (C) Quantitation of cell number (based on *TbZFP3* expression) and relative *PAD1* expression per cell. All data represent the mean and SEM of the analysis of six mice. (D) Representative images of trypanosomes on different days of infection in the first wave of parasitemia. On each day a phase-contrast image and immunofluorescence image of cells stained with antibody to the VSG AnTat1.1 is shown. Scale bars represent 10 μm. See also Figure S2.

The individual parasitemias each comprised an initial major peak extending from day 3 to day 9 after infection; parasite numbers then recrudesced on day 12, whereafter they were sustained (with individual variability) for a further 18 days until the experiment was terminated (Figure 2 and Figure S1 available online). To describe the early phase of infection, cell-cycle status and parasite morphology were scored in each sample over the first 9 days of infection, as was the expression profile of the initiating antigen variant, AnTat1.1, comprising a manual inspection of 9000 cells per parameter (Figure 3 and Figure S2). On days 3 and 4 after infection, infections comprised 99.9% ± 0.1% slender parasites, characterized by their elongate morphology, ovoid nucleus and, where relevant, their observed cell proliferation (Figure 3A and Figure S2A). Although difficult to unambiguously quantitate, intermediate forms began to replace slender forms from day 5 (6.5% ± 1.2%) and by day 6 the population comprised 61% ± 3.8% intermediate forms and 29.5% ± 3.7% slender forms, the remaining cells being emergent stumpy forms (9.6% ± 3%). During days 7 to 8 after infection, slender forms almost completely disappeared (day 7, 2.7% ± 0.54%; day 8, 5.8% ± 1.8%) as stumpy forms accumulated (day 7, 74% ± 3.4%; day 8, 70% ± 2%) although intermediate forms remained in the population (day 8, 24% ± 3%). Cell-cycle analysis confirmed the replacement of proliferative slender forms by stumpy forms (Figure 3B and Figure S2B). Thus, cells with 1 kinetoplast and 1 nucleus (1K1N; i.e., G1, early S phase or G0-arrested cells [Woodward and Gull, 1990]) accumulated from day 5 after infection (day 4, 74% ± 1.2%; day 7, 99.9% ± 0.1%), coincident with the appearance of intermediate forms, whereas 2K1N (G2 and mitotic) and 2K2N (postmitotic) cells decreased from 18.3% ± 0.95% and 7.7% ± 0.4%, respectively, on day 4 to undetectable levels on day 7 after infection, when stumpy cells predominated. Throughout the first peak, VSG AnTat1.1-positive cells comprised >99% of all cells, regardless of their morphology (Figure 3D), until clearance of most of the population by day 9 or 10 after infection (Figure 2).

Having precisely established the key cell-cycle and cellular parameters accompanying differentiation during early infection, we monitored transmission competence by tracking the relative expression of *PAD1* mRNA by qRT-PCR in each mouse on each day of infection. On day 4, *PAD1* mRNA expression was barely detectable (mean relative expression [RE] per cell, 0.6 ± 0.07, with respect to a common baseline standard), but thereafter progressively increased until day 6 (RE = 11.4 ± 0.69), after which no further increase during the first peak of parasitemia was observed (Figure 3C). This revealed that the *PAD1* mRNA expression in stumpy forms was no higher than intermediate forms, unlike its protein expression (Dean et al., 2009). During the chronic phase, in contrast, *PAD1* expression per cell generally tracked the fluctuations in the overall cell number but remained consistently high, close to the peak levels during the first parasitemia (day 12 to day 30 mean RE = 10.28 ± 1.96) (Figure 2 and Figure S1C). Combined, these analyses generated a temporal profile of differentiation events early in infection, whereby the *PAD1* mRNA expression provided an early indicator of differentiation, approximately matching the progression to cell-cycle exit in the population and the appearance of morphologically intermediate forms. In the chronic phase, however, a sustained high level of intermediate and stumpy forms was maintained, with no extended period of low transmission capacity in the parasite populations.

### Mathematic Modeling of Trypanosome within Host Dynamics

**Figure 4:**
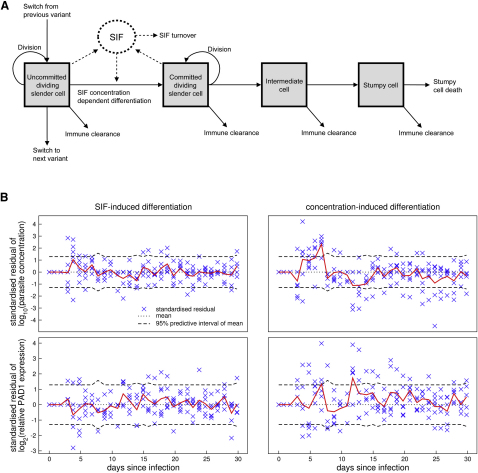
A Mathematical Model for Trypanosome Within-Host Dynamics The diagram represents the following: (A) The developmental steps associated with stumpy formation in vivo as determined by the mathematical model described in Document S2. (B) The adequacy of the fits of the mathematical models to the quantitative data based on either SIF-induced differentiation (left-hand panel) or concentration-induced differentiation (right-hand panel). Crosses are standardized residuals of each data point from each mouse for both parasitemia (upper panel) and *PAD1* expression (lower panel). Poor fit is suggested by outliers that are more than three standard deviations from zero. If the model were true, the mean standardized residual (red line) would lie within its 95% predictive interval 95% of the time. The dashed lines are this interval Bonferroni corrected for multiple tests. See also Figure S3 and Document S2.

The early expression of *PAD1* mRNA in differentiation enabled us to exploit mathematical modeling to reconstruct the key cytological and temporal events during the commitment to generate stumpy forms. It also allowed the contribution of the stumpy transmission stage to the infection to be modeled, a parameter that could be previously estimated based only on limited and subjective data in the literature. Using an adaptive population-based Markov chain Monte Carlo (McMC) method in a Bayesian framework, we fitted the mathematical model to the observed parasite number and *PAD1* expression in each infection (see Document S2). The estimated model parameters suggest that each population is composed of uncommitted slender cells, committed slender cells capable of limited further divisions, cell cycle-arrested intermediate cells, and stumpy cells (Figure 4A). A time-dependent morphological transition through differentiated forms was also incorporated, matching prior in vitro observation or prediction (Tyler et al., 2001; Vassella et al., 1997). The proportion of cells in each developmental stage was considered to be dependent upon their generation rate, comprising the proliferation of noncommitted and committed slender forms, and the loss of each cell type through forward differentiation in response to the proposed cell density signaling factor, SIF (Reuner et al., 1997; Vassella et al., 1997), or, in the case of stumpy forms, cell senescence. In addition, the immune response of the host and immune evasion strategy of the parasite, via antigenic variation, were considered. An initial infection with a population expressing a single antigen type (AnTat 1.1) was assumed. Also, switching between variants was assumed to be sequential with variable switching rates. This assumption is simpler than a nonsequential, hierarchical, switching model (Gjini et al., 2010; Lythgoe et al., 2007) but nonetheless allowed for multiple antigen types to be present simultaneously in the population (refer to Document S2). It therefore provided a parsimonious and biologically relevant approximation of switching. Differential immune-clearance rates of noncommitted slender cells and differentiated cells were included to account for the observed relative sensitivity to antibody lysis of slender and stumpy forms (Engstler et al., 2007; McLintock et al., 1993). The mathematical derivation and biological parameters are described in Document S2.

Residual analysis was used to assess the adequacy of the model fit to the experimentally determined parasite number and *PAD1* expression level (Miller et al., 2010). There were no outlying residuals and the mean of the residuals lay within its Bonferonni corrected 95% predictive interval (Figure 4B). These results suggested an excellent fit to the data. Other models have assumed that differentiation rate is proportional to parasite density (Black et al., 1983; Gjini et al., 2010; Savill and Seed, 2004; Turner et al., 1995) rather than the soluble parasite-derived factor (i.e., SIF) used here. However a cell-density dependent differentiation model poorly fitted the data, as demonstrated by residual analysis (Figure 4B). Indeed, the ratio of the marginalized likelihoods showed the data to be 10^60^ times more likely under the SIF-induced differentiation model than under a parasite density-induced differentiation model, supporting the existence of a soluble factor driving differentiation (Document S2). Parameter estimates (Table 1 and Figure S3) indicated that SIF is generated for 10.7 ± 1.2 hr after commitment to differentiation and that slender cell replication is estimated to take 4.9 ± 0.2 hr, this replication rate agreeing well with in vivo measurement (Seed and Black, 1997; Vassella et al., 1997). Given that committed slender cells are estimated to have a life span of 14.6 ± 0.7 hr, these estimates indicate that SIF production occurs only in slender cells and ceases prior to the terminal division that generates intermediate forms. The turnover of SIF in vivo is estimated to be 0.3 ± 0.04 hr^−1^, a half-life of approximately 2.3 hr (Table 1).

**Figure 5:**
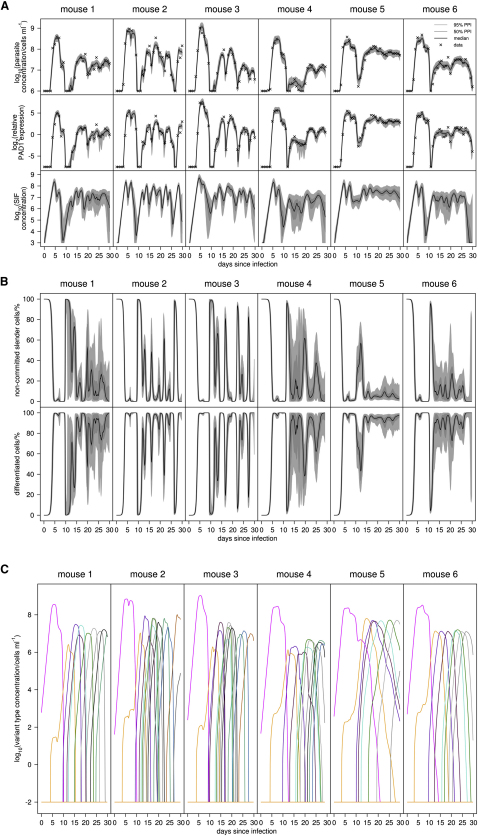
Analysis of the Model Predictions and Data Values for Trypanosome Chronic Infections (A) Model fits to parasite density and relative total *PAD1* expression in the population (crosses, data; dark gray shading, 95% posterior predictive interval [PPI] of dynamics; light shading, 50% PPI; line, median dynamics). The model's posterior prediction of SIF concentration dynamics with 95% and 50% intervals is also shown. (B) The model's posterior prediction of percentage of noncommitted slender cells and differentiating cells (committed slender, intermediate and stumpy cells) with 95% and 50% intervals. (C) Predicted median dynamics of the succession of antigenic variants in each mouse over 30 days of infection. See also Figure S4 and Document S2.

Analysis of the model also emphasized the dominance of intermediate and stumpy forms in chronic infections (Figure 5). Thus, differentiated cells were predicted to comprise the majority of all infections beyond the first wave of parasitemia, matching the experimental observation that *PAD1* expression levels remained elevated (Figures 2, 5A, and 5B). Confirming this, a blinded post-hoc analysis of the infection in three mice (M2, M3, and M5) on days 15 to 17 (Figure S4) demonstrated that the proportion of slender cells varied from 3.2%–27% (mean, 14.5% ± 0.9%), with intermediate and stumpy cells representing 73%–97% (mean, 85.5% ± 0.9%) of the population.

An important consequence of the low frequency of slender cells in chronic infections is a low rate of productive antigen variation, since intermediate and stumpy forms do not replicate (Reuner et al., 1997) and cannot contribute to new populations of antigenic variants. This was borne out by analysis of the predicted number of variants in mice that differed in the number of differentiated parasites throughout infection (Figure 5C). Thus, mouse 2, which sustained more slender cells throughout infection, was predicted to exhibit 12.5 ± 1.7 variants in 30 days whereas mouse 5, which sustained a high level of stumpy forms, was predicted to exhibit 8 ± 2.0 variants. In both cases the initial immune activation against the infecting antigen type (AnTat1.1) was similar, being 142 ± 4.78 hr and 152 ± 5.98 hr respectively (Figure S3). Although the precise number of antigenic variants may be expanded by a hierarchical rather than sequential switching model, it is clear that maintaining abundant transmissible forms reduces the rate of antigen usage per unit time. We conclude that differentiated parasites are the major component of long term infections, this restricting the number of antigen types expressed.

## Discussion

The factors determining the probability of transmission are important contributors to shaping the life-history evolution of parasites. Although many studies of trypanosome within-host growth have focused on the parasite's immune evasion dynamics, these analyses have been limited by an absence of definitive markers that discriminate transmissible stumpy forms, such that the contribution of cell-type differentiation could only be estimated. Moreover, no previous studies have quantitatively assessed the proportion of this transmission stage in long-term chronic infections, nor determined its prevalence early and late in infection. In consequence, the contribution of cell differentiation to trypanosome infection dynamics has remained unknown despite over 100 years of study. The results in this study provide a robust quantitative analysis of within-host differentiation, generating a detailed picture of chronic infections with African trypanosomes. Further, mathematical interpretation of the observed biological data has produced an in vivo analysis of the establishment phase of infection and provided a map of the associated developmental events. This involves a commitment of slender forms to differentiation, followed by two to four (3.0 ± 0.59) further cell divisions before cell-cycle exit. *PAD1* mRNA expression begins upon differentiation to intermediate forms, revealing this to be a better marker for differentiation than cell morphology and, indeed, morphological analyses provided a relatively poor fit in our mathematical analysis. *PAD1*-expressing cells then undergo morphological transformation to stumpy forms, with an anticipated lifespan of 49.9 ± 5.7 hr. This matches expectation (Black et al., 1982; Savill and Seed, 2004; Turner et al., 1995), as does the observation that immune clearance was predicted to be more effective for slender cells than stumpy cells (Engstler et al., 2007; McLintock et al., 1993), which were predicted to be lost through cell aging. Importantly, statistical analysis has also generated overwhelming support for the existence of a parasite-derived soluble signal governing differentiation and derived parameter predictions for its production and turnover in vivo. Clearly, these parameters may differ quantitatively in different hosts, affecting the threshold at which stumpy-formation is initiated, but the overall hierarchy of events would be preserved. Moreover, without identification of the proposed signaling factor, it is not possible to discriminate whether the signaling mechanism requires group cooperation (quorum sensing) to coordinate differentiation in the population in response to cell density or a diffusion/efficiency-sensing mechanism that accounts for the spatial distribution of parasites within the mammalian host (Hense et al., 2007).

Analyses of the chronic phase of infection provided some surprising insights into the trade off between parasite virulence and transmission. First, these results demonstrate that trypanosomes invest heavily in sustaining high levels of transmissible forms in long-term infections, a finding consistent with observations in cattle (Van den Bossche et al., 2005) and monkeys (Robertson, 1912) and perhaps selected for by the inefficient vectorial capacity of tsetse flies in the field. Second, a direct consequence of this investment in transmission stages is an inevitable limitation in the overall frequency of antigenic variation in the parasite population, since only slender cells can generate new variant populations. As slender cells in natural pleomorphic infections switch at high frequency (Turner and Barry, 1989), we propose that this produces a scenario where there is rapid antigenic switching during the early phase of infection when slender cells dominate, allowing parasites to probe the immune system for prior exposure to some antigen types. This could help the parasites to establish in a new host and limit the impact of potential herd immunity or super-infection (Marcello and Barry, 2007). Once established, however, we expect the switching potential in the population to be reduced as transmission stages dominate and slender cells form a small proportion of the total parasite load. We suggest that this could both limit the rapid exposure of the parasite's antigen repertoire to the immune system and prevent the host immune system being overwhelmed by a multitude of new variants (Gjini et al., 2010). Although this population structure would not affect the hierarchy of switching or the ordered procession of antigen types expressed by the minor slender population (Gjini et al., 2010; Lythgoe et al., 2007; Marcello and Barry, 2007; Morrison et al., 2005), their rapid switching could contribute to the maintenance of the infection. This is particularly relevant in long-term infections, where there is an increasing pressure to activate VSG genes not previously expressed, or to assemble functional VSG gene mosaics. Elegantly, this population structure could balance the need to optimize the probability of transmission, while ensuring that new variants are generated at a frequency that ensures infection chronicity without overwhelming the host.

The balance between the frequency of the generation of new antigenic variants and their control by the immune system is a central component of models of antigenic variation and infection chronicity in many systems (Deitsch et al., 2009; Frank and Barbour, 2006; Lange and Ferguson, 2009), such as bacteria (*Anaplasma, Borrelia* [Palmer et al., 2009]), *Plasmodium* (Gupta, 2005; Recker et al., 2004), and African trypanosomes. Deciphering this complexity requires an understanding of the different components of the infection dynamics, such as the determinants of antigen expression hierarchy (e.g., the sequence relationships between, and the group sizes of, related antigen types) (Barbour et al., 2006; Morrison et al., 2005; Palmer and Brayton, 2007), the specificity of the immune response to related and unrelated antigens (Recker and Gupta, 2006; Recker et al., 2004), and the trade-off between virulence and chronicity for transmission probability (Lange and Ferguson, 2009). Each of these components is multiply linked such that predicting their impact requires a detailed experimental analysis of their contribution and a mathematical analysis of their interactions when combined, which in turn can generate an evolutionary understanding of the infection. Our study has provided the experimental framework for dissecting the contribution of transmission stages to the within-host dynamics of trypanosome infection, which in future must integrate with similarly detailed analyses of antigen switching and immune responses to provide a comprehensive understanding of infection chronicity. Such studies will allow the identification of the important parameters that contribute to trypanosome maintenance in reservoir hosts and transmission in the field. Most importantly, this enables the possible consequences of therapeutic efforts directed against either proliferative slender forms or transmission blocking strategies targeting intermediate and stumpy forms, to be predicted and control measures optimized.

## Experimental Procedures

### Trypanosome Infection

*Trypanosoma brucei* AnTat1.1 were infected into age-matched male MF1 and monitored by microscopy (Herbert and Lumsden, 1976). Cell morphology, cell-cycle analysis, and VSG staining were carried out as described previously (Tasker et al., 2000).

### RNA Extraction

Ten microliters of blood was collected with a capillary tube (Camlab DMP010) and transferred into 20 μl Nucleic Acid Purification Lysis Solution (ABI 4305895) with 10 μl prechilled 1× PBS, then mixed and stored at −80°C. RNA was extracted using an ABI Prism 6100 Nucleic Acid PrepStation with the “RNA Blood-DNA” program, with vacuum removal of reagents between steps. Wells were prewet with 40 μl RNA Purification Wash Solution 1 (ABI 4305891), 40 μl lysate was added, and then washed with 650 μl RNA Purification Wash Solution 1 followed by 650 μl RNA Purification Wash Solution 2 (ABI 4305890). Fifty microliters of AbsoluteRNA Wash Solution (ABI 4305545) was then incubated for 15 min, after which 400 μl RNA Purification Wash Solution 2 was incubated for 5 min. One hundred microliters of Nucleic Acid Purification Elution Solution was added and RNA eluted into a MicroAmp Optical 96-well Reaction Plate (ABI 4306737). Contaminating genomic DNA was removed with an Ambion TURBO DNA-free kit (Applied Biosystems AM1907).

### cDNA Preparation and RT-PCR

Complementary DNA (cDNA) was produced with the ABI High-Capacity cDNA Reverse Transcription kit (ABI 4368813) according to manufacturer's instructions and then amplified on a ABI StepOnePlus RT-PCR machine. Melt curve analysis verified there was only one amplification product. For the constitutively expressed posttranscriptional regulator *TbZFP3* (Paterou et al., 2006), a standard curve was used for quantification of parasite number. For *PAD1* the ΔΔCT method was used for relative quantification, using *TbZFP3* as an internal control. The data were analyzed with the ABI StepOne software version 2. Primers used for the amplification of *PAD1* cDNA were 5′-GACCAAAGGAACCTTCTTCCT-3′ and 5′-CACTGGCTCCCCTAAGCT-3′. For *TbZFP3*, the primers 5′- CAGGGGAAACGCAAAACTAA-3′ and 5′-TGTCACCCCAACTGCATTCT-3′ were used.

### Mathematical Modeling

Details are provided in Document S2.

## Figures and Tables

**Table 1:** Key Parameters Associated with Stumpy Formation In Vivo as Determined by the Mathematical Model Described in Document S2

Parameter	Mean (±SEM)
Slender cell cycle	4.9 ± 0.2 hr
Committed slender cell life span	14.6 ± 0.7 hr
Stumpy cell life span	49.9 ± 5.7 hr
Total committed cell life span	70.0 ± 5.7 hr
Committed slender replications	3.0 ± 0.2
Committed cell SIF production	10.7 ± 1.2 hr
Differentiation rate	1.6 E-09 ± 2.3 E-10/hr
SIF removal rate	0.30 ± 0.04/hr
Time of initial immune activation	142.5 ± 6.9 hr
Duration of *PAD1* rise	14.4 ± 1.5 hr
log10 initial parasite concentration	2.3 ± 0.2/ml
Number of variant types	10.5 ± 0.7

See also Document S2.

## Supplemental Information

Document S1. Supplemental Experimental Procedures and Four Figures

Document S2. Mathematical Model Description, Related to the Experimental Procedures, Figures 4 and 5, and Table 1
