# Supplementary material for: Transmission Stages Dominate Trypanosome Within-Host Dynamics during Chronic Infections
Source: Cell Host Microbe. 2011 Apr 21;9(4):310–8. doi: 10.1016/j.chom.2011.03.013 (PMC3094754; doi:10.1016/j.chom.2011.03.013)
Supplement: Document S1. Supplemental Experimental Procedures and Four Figures [file mmc1.pdf]

Cell Host & Microbe, ***Volume 9***

Supplemental Information

Transmission Stages Dominate Trypanosome Within-Host Dynamics during Chronic Infections

Paula MacGregor, Nicholas J. Savill, Deborah Hall, and Keith R. Matthews

Supplemental Experimental Procedures

Trypanosome Infection

Trypanosoma brucei AnTat1.1 were infected into age-matched male MF1 and monitored by microscopy (W. J. Herbert, W. H. Lumsden, *Exp Parasitol* **40**, 427.) Cell morphology, cell-cycle analysis and VSG staining were carried out as described previously (M. Tasker, J. Wilson, M. Sarkar, E. Hendriks, K. Matthews, *Mol Biol Cell* **11**, 1905, 2000).

Quantitative RT-PCR from whole blood

Sample preparation
From an infected mouse, 10µl of whole blood was collected using a 10µl capillary tube (Camlab DMP010) and immediately transferred into 20µl Nucleic Acid Purification Lysis Solution (ABI 4305895) with 10µl 1x PBS, pre-chilled on ice. The solution was mixed by flicking the tube and was stored at -80°C until required.

For the purposes of production of a standard curve, the concentration of parasites in the blood was required to be known. In this case, slender or stumpy parasites were harvested and purified from a mouse infection. The cells were counted and a known number of cells were centrifuged at 800g for 10 minutes to pellet the cells. The parasites were then resuspended in a known volume of blood such that the final concentration of parasites per ml blood could be calculated. This blood was divided into 10µl aliquots added to 30µl lysis mix (see above) before being stored at -80°C. These standard curve samples were processed in the same manner as experimental samples.

RNA extraction from whole blood
RNA was extracted using an ABI Prism 6100 Nucleic Acid PrepStation according to manufacturer's instructions. The platform takes 96-well RNA purification trays (ABI 4305673) to which the sample and appropriate washes are applied, and pulls the flow-through into the waste collection by applying a vacuum across the wells. A splashguard (ABI 4311758) was used in order to prevent contamination between wells. All RNA extractions were carried out using the 'RNA Blood-DNA' programme. For this, wells were pre-wet with 40µl of RNA Purification Wash Solution 1 (ABI 4305891) and then 40µl of lysate was added to each well. An 80% vacuum was applied to the wells for 3 minutes. Wells were washed with 650µl of RNA Purification Wash Solution 1 and the wash was removed by applying an 80% vacuum to the wells for 3 minutes. Wells were

then washed with 650µl of RNA Purification Wash Solution 2 (ABI 4305890) and the wash was removed by applying an 80% vacuum to the wells for 3 minutes. Taking care to apply directly to the bottom of the well, 50µl of AbsoluteRNA Wash Solution (ABI 4305545) was added to the wells and incubated for 15 minutes. The AbsoluteRNA Wash Solution contains a DNase which should remove genomic DNA from the sample. Due to the labile nature of DNase this was defrosted on ice with care taken not to destroy the DNase mechanically. Without removing the AbsoluteRNA Wash Solution, 400µl of RNA Purification Wash Solution 2 was added and this was incubated for 5 minutes before removal by applying an 80% vacuum to the wells for 3 minutes. The well was washed twice more with RNA Purification Wash Solution 2, firstly with 650µl and then 400µl, each time removing the wash by applying an 80% vacuum to the wells for 3 minutes. A 90% pre-elution vacuum was applied for 5 minutes to remove any remaining wash solution before moving the purification try from the waste collection position to the sample collection position. To elute the sample, 100µl of Nucleic Acid Purification Elution Solution was added to each well and a 20% vacuum was applied to the wells for 2 minutes. Samples were eluted into a MicroAmp Optical 96-well Reaction Plate (ABI 4306737), sealed with an adhesive film (ABI 4311971) and stored at -80°C until use.

Treatment of RNA with TURBO DNase
An additional DNase treatment was added after RNA extraction using an Ambion TURBO DNA-free kit (Applied Biosystems AM1907) according to manufacturers' instructions. A typical 50µl reaction is shown below.

DNase reaction: 5µl 10X TURBO DNase Buffer
44µl RNA
1µl TURBO DNase

The reaction was mixed gently and incubated at 37°C for 30 minutes before deactivating the DNase with 0.1 volume of DNase Inactivation Reagent (in this case, 5µl). The reaction was mixed well and incubated for 5 minutes with occasional mixing to disperse the DNase Inactivation Reagent. The sample was centrifuged at 4000g for 1.5 minutes and the RNA was transferred to a fresh tube. The RNA was stored at -80°C until use and care is taken to avoid repeated freeze thaw cycles.

cDNA production
cDNA was produced using the ABI High-Capacity cDNA Reverse Transcription kit (ABI 4368813) according to manufacturer's instructions. A typical 20µl reaction is shown below.

RT reaction:	10µl Turbo DNase-treated RNA (diluted 1:2 in dH_2O)
	2µl 10x RT Buffer
	0.8µl 25x dNTP Mix (100mM)
	2µl 10x RT Random Primers
	1µl MultiScribe Reverse Transcriptase
	3.2µl Nuclease-free dH_2O (Promega P1195)
Reaction Conditions:	25°C 10 minutes
	37°C 120 minutes

The reaction was carried out in 0.2ml PCR tubes (Axygen 321-10-051) in a Thermo Electron Corporation PCR SPRINT thermal cycler. Controls which replace the reverse transcriptase with dH_2O were used to ensure that there was not any contamination from DNA in the sample. The cDNA was stored at -20°C until required.

Quantitative RT-PCR

Quantitative RT-PCR (qRT-PCR) was carried out on an ABI StepOnePlus RT-PCR machine to amplify either *PAD1* or *Tb*ZFP3 mRNA. Reactions were carried out in 25µl volumes, as detailed below. A melt curve was added to the end of each qRT-PCR to ensure that there was only one amplification product. For *Tb*ZFP3, a standard curve was for absolute quantification of parasite number. For PAD1 the ΔΔCT method of data analysis was used for relative quantification, using *Tb*ZFP3 as an internal control. The data was analysed using the ABI StepOne software version 2.

PAD1 qRT-PCR Reaction: 12.5µl Power SYBR Green PCR Master Mix (ABI 4367659)
0.75µl 10µM Primer 24
0.75µl 10µM Primer 25
4µl dH_2O
7µl cDNA diluted 1/10

ZFP3 qRT-PCR Reaction: 12.5µl Power SYBR Green PCR Master Mix (ABI 4367659)
2.25µl 10µM Primer 17
2.25µl 10µM Primer 19
1µl dH_2O
7µl cDNA diluted 1/10

Reaction Conditions: 95°C 10 minutes
40 x [95°C for15 seconds, 60oC for 1 minute]
+ Melt curve

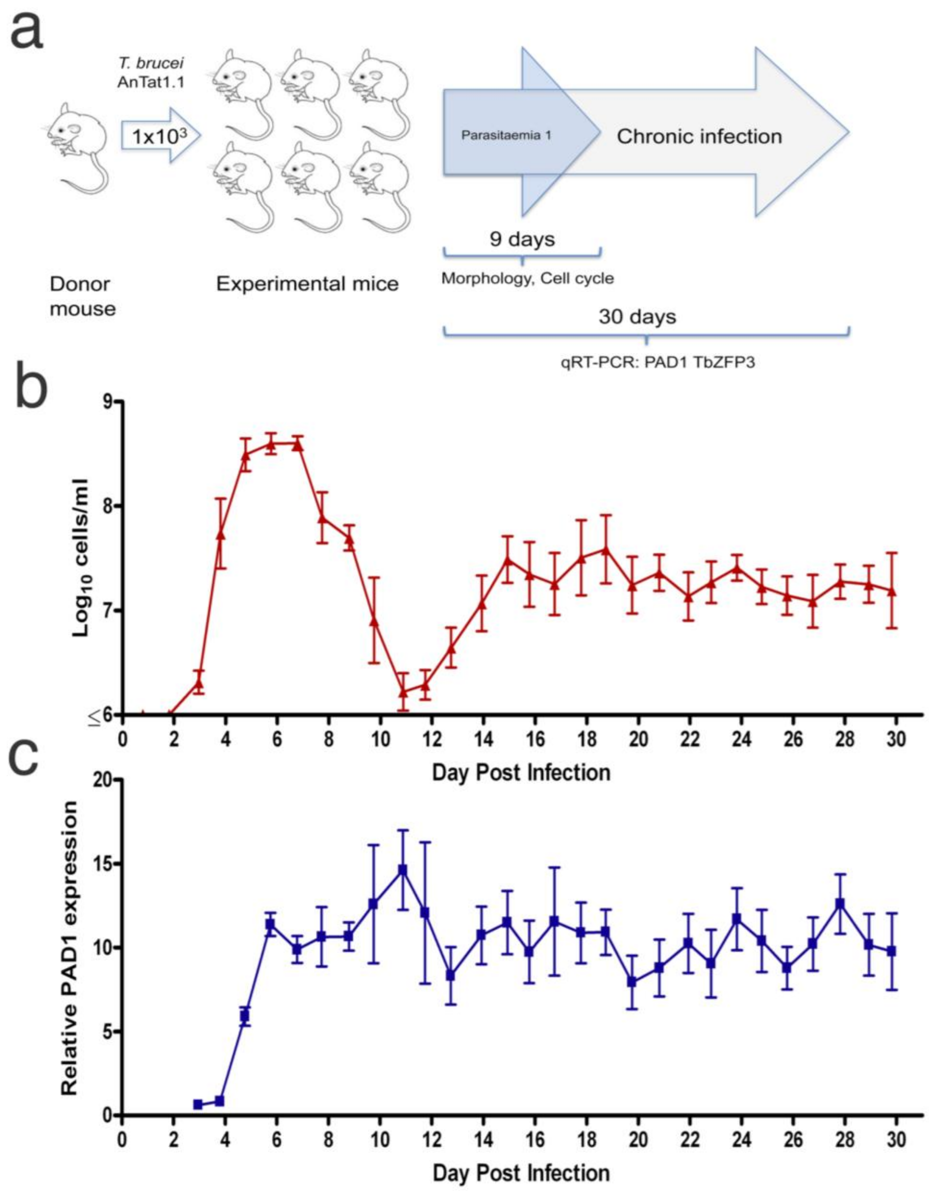

Figure S1. Related to Figure 2

(a) Schematic representation of the experimental design, (b) quantification of cell number based on the expression of *Tb*ZFP3 mRNA, (c) quantitation of relative *PAD1* mRNA expression, values being expressed relative to a common standard in each assay (mouse 5, day 4 mRNA). In (b) and (c), values represent the mean and standard error from six mice on each day of infection. Individual data sets are provided in Figure 1.

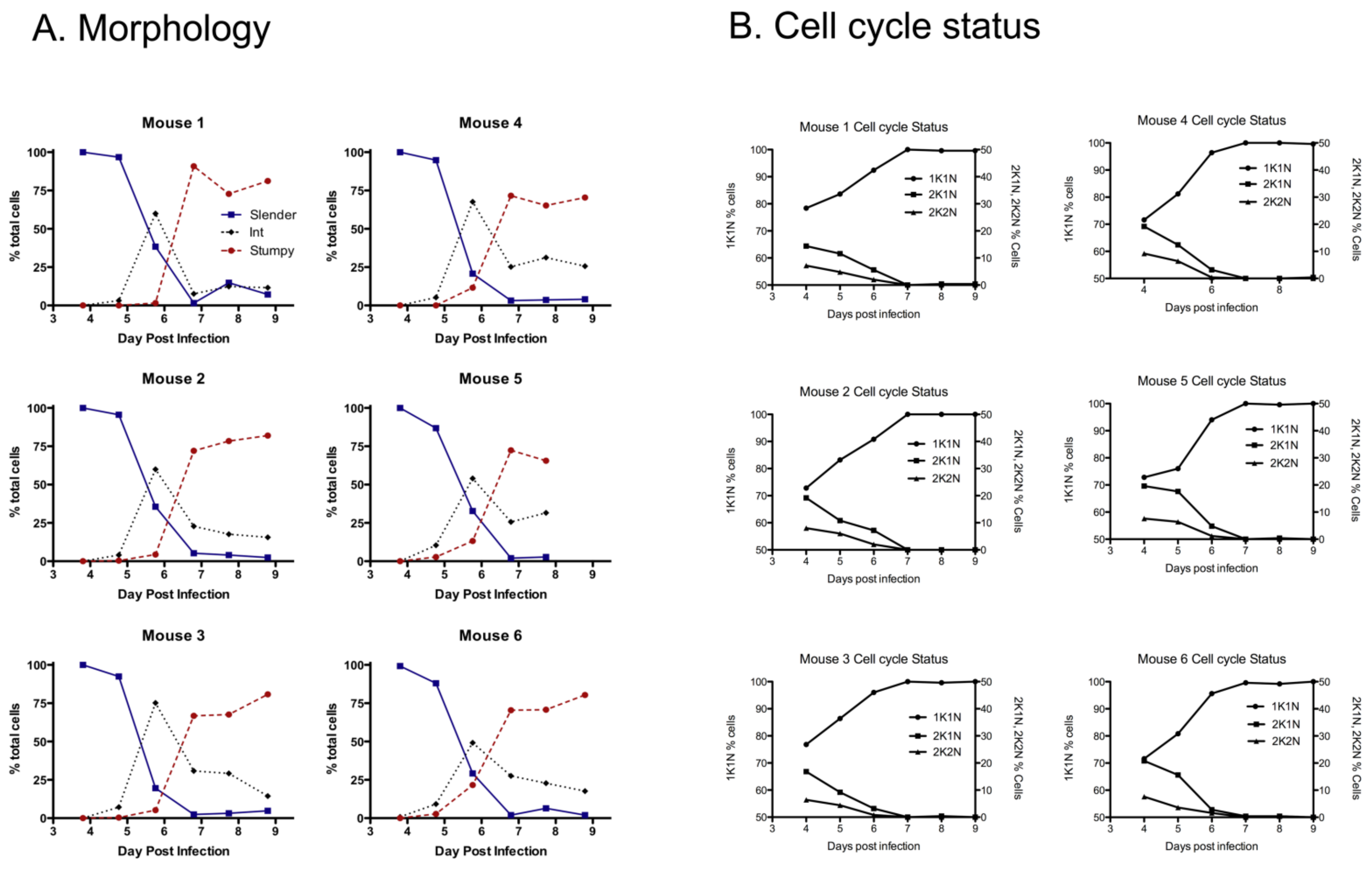

Figure S2. Related to Figure 3

a. Analysis of individual mice during days 3 to 9 post infection for the morphology of cells in each population (i.e. slender, intermediate or stumpy form). The data represents an analysis of 250 cells per time point in each mouse.

b Analysis of individual mice during days 3 to 9 post infection for the cell cycle stage of the observed parasites. The data represents an analysis of 250 cells per time point in each mouse.

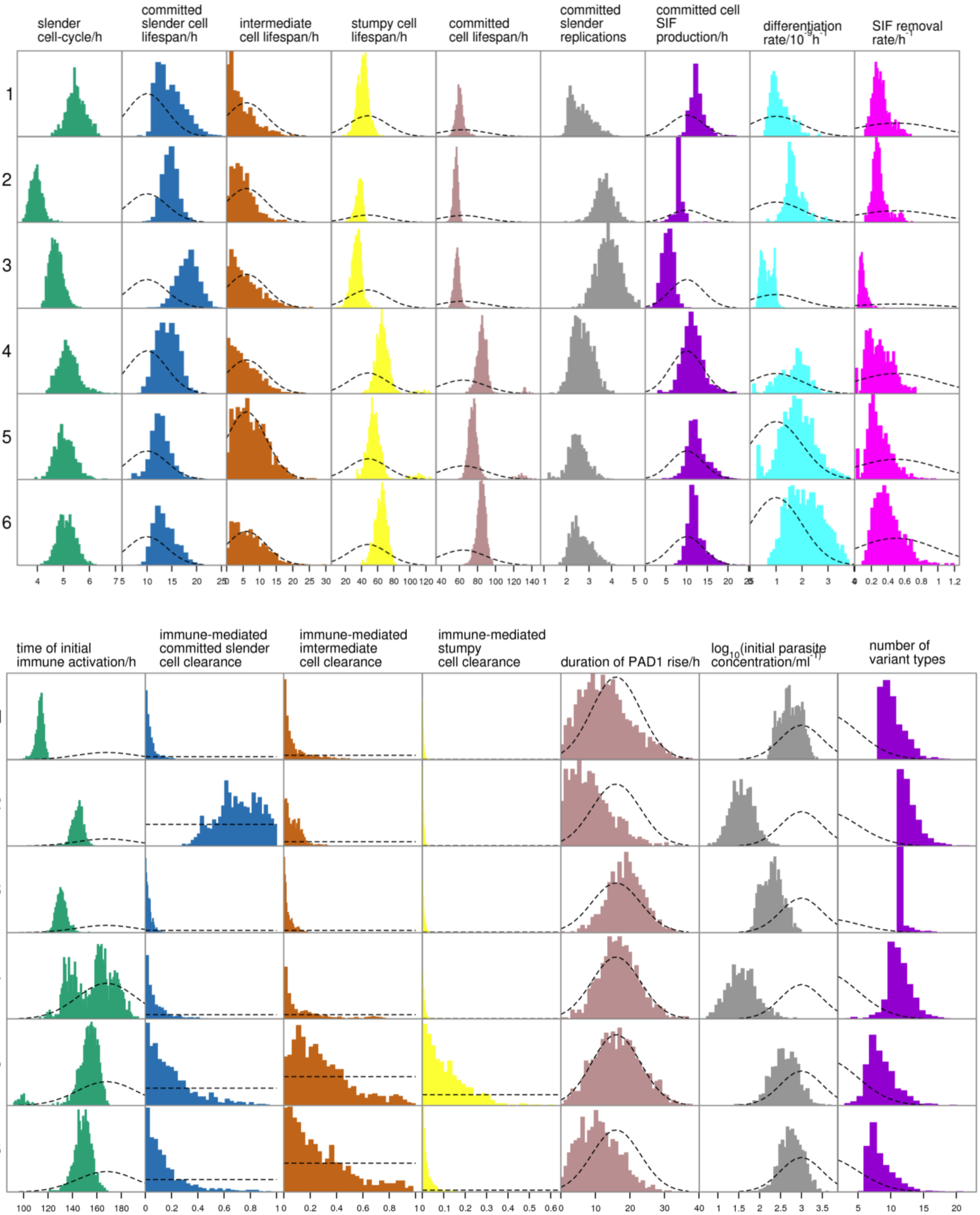

Figure S3. Related to Figure 4

Parameter prior distributions (dashed lines where appropriate) and posterior distributions (histograms) for each mouse.

% of each cell-cycle configuration (n=250)

Mouse 2

Day	Slender			Int	Stumpy	Total
	1K1N	2K1N	2K1N	1K1N	1K1N	
15	10.8	1.2	0.4	32	55.6	100
16	8.4	0.8	0	27.6	63.2	100
17	15.2	2.8	0.8	33.6	47.6	100

Mouse 3

Day	Slender			Int	Stumpy	Total
	1K1N	2K1N	2K1N	1K1N	1K1N	
15	17.6	4.4	1.2	25.6	51.6	100.4
16	14.8	4.8	2.4	26.8	50.8	99.6
17	22.8	3.2	1.2	28.4	44.4	100

Mouse 5

Day	Slender			Int	Stumpy	Total
	1K1N	2K1N	2K1N	1K1N	1K1N	
15	9.2	1.6	0	32.4	56.8	100
16	5.2	0.4	0.4	32.8	61.2	100
17	3.2	0	0	22.4	74.4	100

Figure S4. Related to Figure 5

Proportion of cells with 1 kinetoplast, 1 nucleus (1K1N), 2 kinetoplasts**,** 1 nucleus (2K1N) and 2 kinetoplasts, 2 nuclei (2K2N) on day 15, day 16 and day 17 post infection in mice 2, 3 and 5. On each day, 250 cells were scored for their cell-cycle stage and cellular morphology. Values are presented as a percentage of the total population scored.
