# Supplementary material for: Transmission Stages Dominate Trypanosome Within-Host Dynamics during Chronic Infections
Source: Cell Host Microbe. 2011 Apr 21;9(4):310–8. doi: 10.1016/j.chom.2011.03.013 (PMC3094754; doi:10.1016/j.chom.2011.03.013)
Supplement: Document S2. Mathematical Model Description, Related to the Experimental Procedures, Figures 4 and 5, and Table 1 [file mmc2.pdf]

Supplementary Material: Transmission stages dominate trypanosome within-host dynamics during chronic infections

Paula MacGregor, Nicholas J. Savill, Deborah Hall and Keith R. Matthews

1 The model

The model is constructed as follows. Let the concentration of non-committed slender cells of variant type v at time t be $l_v(t)$. The initial infection by variant type 1 is at time $t = 0$. Switching between variants is sequential, i.e., variant v switches to variant $v + 1$ and no other. This is different than other models [3] where one variant may switch to a number of different variants. Although this assumption was introduced to reduce the number of parameters associated with the model, it was found to generate simultaneous expression of multiple variants. Hence, although a simplified representation, a good match to biological observation is generated.

Non-committed slender cells replicate at rate α (i.e., a cell-cycle time of $\frac{\ln 2}{\alpha}$). They are cleared by a time-dependent, variant-specific immune response at rate $r_v(t)$. They become committed to differentiate at rate $\beta f(t)$, where $f(t)$ is SIF concentration, and β a constant of proportionality. Switching from variant v to variant $v + 1$ is at variant specific rate ω_v. Thus the differential equations that describe the dynamics of each non-committed slender variant are

$$\frac{d}{dt} l_v(t) = (\alpha - \omega_v - \beta f(t) - r_v(t)) l_v(t) + \begin{cases} 0 & \text{if } v = 1 \\ \omega_{v-1} l_{v-1}(t) & \text{if } v = 2, \ldots, \nu \end{cases} \quad (1)$$

where ν is the total number of variant types that appear in a mouse during the experiment. Let $L(t)$ be the total concentration of non-committed slender cells; it is given by the sum over all variant types

$$L(t) = \sum_{v=1}^{\nu} l_v(t) \quad (2)$$

Let the age of differentiated cells since becoming committed to differentiation be a, and let $d_v(a, t)$ be the age distribution of differentiated cells of variant type v at time t.

Differentiated cells fall into three classes: replicating, committed slender cells, non-replicating intermediate cells and non-replicating stumpy cells. All committed cells of variant type v are cleared by the immune system at a rate proportional to the clearance rate of non-committed slender cells of the same type, i.e., $r_v(t)$. Thus committed slender cells are cleared by the immune response at rate $\delta_c r_v(t)$. At age τ_c they become intermediate cells. Intermediate

cells are cleared at rate $\delta_i r_v(t)$. They become stumpy cells at age τ_i. Stumpy cells are cleared at rate $\delta_s r_v(t)$. They die at age τ_s. Committed slender cells replicate with rate α. Thus the partial differential equations that describe the dynamics of the age distribution of each differentiated variant type v are

$$\frac{\partial}{\partial t}d_v(a,t)+\frac{\partial}{\partial a}d_v(a,t)=-d_v(a,t)\times\begin{cases}\delta_c r_v(t)-\alpha & \text{if } 0\le a<\tau_c\\ \delta_i r_v(t) & \text{if } \tau_c\le a<\tau_i\\ \delta_s r_v(t) & \text{if } \tau_i\le a<\tau_s\end{cases} \tag{3}$$

The boundary conditions on these equations are determined by differentiation of non-committed slender cells into age $a=0$, i.e., $d_v(0,t)=\beta f(t)l_v(t)$, and stumpy death at age τ_s, i.e., $d_v(\tau_s,t)=0$.

Let $D(a,t)$ be the age distribution of differentiated cells of all variant types at time t. It is given by the sum over all variants

$$D(a,t)=\sum_{v=1}^{\nu}d_v(a,t) \tag{4}$$

Let $C(t)$ be the total concentration of committed slender cells, let $I(t)$ be the total concentration of intermediate cells, let $S(t)$ be the total concentration of stumpy cells, and let $T(t)$ be the total concentration of cells. These are given by

$$C(t)=\int_0^{\tau_c}D(a,t)da \tag{5}$$

$$I(t)=\int_{\tau_c}^{\tau_i}D(a,t)da \tag{6}$$

$$S(t)=\int_{\tau_i}^{\tau_s}D(a,t)da \tag{7}$$

$$T(t)=L(t)+C(t)+I(t)+S(t) \tag{8}$$

SIF is produced by non-committed slender cells and committed cells up to age τ_f at the rate of 1 unit of SIF per hour. SIF is lost at rate γ. Thus the differential equation describing the dynamics of SIF concentration is

$$\frac{d}{dt}f(t)=L(t)+\int_0^{\tau_f}D(a,t)da-\gamma f(t) \tag{9}$$

PAD1 is only expressed in differentiated cells. It is scaled relative to the PAD1 expression of mouse 5 on day 4. It rises linearly from 0 up to a level ρ_1 in cells from age 0 to age τ_p respectively. It then rises or falls linearly to a level ρ_2 in cells just before they die at age τ_s. Let $p(a)$ be the relative PAD1 expression in a differentiated cell of age a, it is given by

$$p(a)=\begin{cases}\rho_1\frac{a}{\tau_p} & \text{if } 0\le a<\tau_p\\ \rho_1+(\rho_2-\rho_1)\frac{a-\tau_p}{\tau_s-\tau_p} & \text{if } \tau_p\le a<\tau_s\end{cases} \tag{10}$$

Let $P(t)$ be the total relative PAD1 expression per ml of blood at time t. It is found by multiplying the concentration of cells of age a by their relative PAD1 expression and then integrating over all ages, i.e.,

$$P(t)=\int_0^{\tau_s}p(a)D(a,t)da \tag{11}$$

The immune response against trypanosomes is multifactorial and highly complex, and only qualitatively understood at best. A detailed mathematical model of the immune response is, therefore, of little use when no data is available to fit to. Instead, we use a single variable $r_v(t)$, that determines the clearance rate of each variant type v. We assume that the immune response against variant type v is activated at a rate ψ by replicating slender cells of variant type v, and self-enhances at rate ϕ. We also assume that the initial immune response to the first variant is only activated after a certain time τ_r. The differential equations that describe the immune-mediated clearance rates of each variant type v are

$$\frac{d}{dt}r_v(t) = \begin{cases} 0 & \text{if } t < \tau_r \\ \phi r_v(t) + \psi l_v(t) & \text{if } t \geq \tau_r \end{cases} \tag{12}$$

Naive mice are infected with non-committed slender cells of variant type 1 at a concentration λ. Therefore the initial conditions are $l_1(0) = \lambda$, $l_v(0) = 0$ for $v = 2, \ldots, \nu$, $d_v(a, 0) = 0$ for all v and a, $r_v(0) = 0$ for all v, and $f(0) = 0$. These imply $L(0) = \lambda$, $D(a, 0) = 0$ for all a and $C(0) = I(0) = S(0) = T(0) = P(0) = 0$.

All variables, functions and parameters are listed in Table 1.

1.1 Cell-Concentration induced differentiation

Several papers [10, 11, 3] have suggested that differentiation is cell-concentration induced rather than SIF induced. We tested this hypothesis by removing $f(t)$ from the model and replacing $\beta f(t)$ by $\beta' T(t)$ in Equation 1, i.e.,

$$\frac{d}{dt}l_v(t) = \alpha - \omega_v - \beta' T(t) - r_v(t))l_v(t) + \begin{cases} 0 & \text{if } v = 1 \\ \omega_{v-1}l_{v-1}(t) & \text{if } v = 2, \ldots, \nu \end{cases} \tag{13}$$

and by changing the boundary condition $d_v(0, t) = \beta' T(t) l_v(t)$.

2 Model fitting, parameter estimation and hypothesis testing

2.1 Likelihood

The measurement errors in the ZFP3 and PAD1 Ct values are assumed to be normally distributed. In other studies, the standard deviation of the errors in $\log_{10}$-transformed concentrations derived from qPCR is approximately 0.20 [5, 6]. This means that the standard errors in Ct values (which are on a $\log_2$ scale) are approximately $0.2 \log_2 10$ which equals 0.66.

The model is fitted to each mouse's $\log_{10}$-transformed parasite concentrations and relative Ct-values for PAD1 expression. Let $\sigma_L = 0.2$ be the standard deviation in the normally distributed errors of $\log_{10}$-transformed parasite concentrations, and let $\sigma_P = 0.66$ be the standard deviation in the normally distributed errors of $\log_2$-transformed PAD1 expression.

For particular values of the model parameters, the model is solved numerically for each mouse using a discrete formulation of the model with a time-step of 1h. In order to quantify the fit of the model with these parameters to the

**Independent variables**		
t	time since infection	h
a	age of differentiated cells	h
**Dependent variables**		
$l_v(t)$	concentration of non-committed slender cells of variant type v	cells ml^{-1}
$d_v(a,t)$	age distribution of differentiated cells of variant type v	cells ml^{-1}h^{-1}
$r_v(t)$	immune-mediated clearance rate of replicating slender cells of variant type v	h^{-1}
$f(t)$	SIF concentration	dimensionless
$L(t)$	total concentration of non-committed slender cells	cells ml^{-1}
$C(t)$	total concentration of committed slender cells	cells ml^{-1}
$I(t)$	total concentration of intermediate cells	cells ml^{-1}
$S(t)$	total concentration of stumpy cells	cells ml^{-1}
$T(t)$	total concentration of all cells	cells ml^{-1}
$D(a,t)$	age distribution of differentiated cells	cells ml^{-1}h^{-1}
$P(t)$	total relative PAD1 expression per ml of blood	ml^{-1}
**Functions**		
$p(a)$	relative PAD1 expression of cells of age a	cells^{-1}
**Parameters**		
ν	number of variant types	dimensionless
α	replication rate of slender cells	h^{-1}
β	differentiation rate	h^{-1}
γ	SIF removal rate	h^{-1}
δ_c	proportionality constant for immune-mediated clearance of committed slender cells	dimensionless
δ_i	proportionality constant for immune-mediated clearance of intermediate cells	dimensionless
δ_s	proportionality constant for immune-mediated clearance of stumpy cells	dimensionless
τ_c	lifespan of committed slender cells	h
τ_i	lifespan of intermediate cells	h
τ_s	lifespan of stumpy cells	h
τ_p	duration of initial rise in PAD1 expression	h
τ_f	duration of SIF production of committed cells	h
τ_r	time until initial activation of immune response	h
ρ_1	relative PAD1 expression of cells age τ_p	cells^{-1}
ρ_2	relative PAD1 expression of cells age τ_s	cells^{-1}
ϕ	self-induced growth rate of immune response	h^{-1}
ψ	committed slender cell-induced growth rate of immune response	h^{-1}
λ	initial concentration of variant type 1	cells ml^{-1}
ω_v	switch rate from variant type v to variant type $v+1$	h^{-1}

Table 1: Variables, functions and parameters used in the model

data we proceed by calculating the log-likelihood of the model solution at each data point. The value of the likelihood is given by the normal probability density function because the errors are normally distributed. So, for an observed parasite concentration $L_{\text{data}}(t)$, at time t above the detection limit (about 10^6 parasites ml^{-1}), the log-likelihood is proportional to

$$\ln\left(\phi\left(\frac{\log_{10} L_{\text{data}}(t) - \log_{10} L_{\text{model}}(t)}{\sigma_L}\right)\right) \tag{14}$$

where $\phi(x)$ is the normal probability density function. For an observed parasite concentration below the detection limit at time t the log-likelihood is proportional to

$$\ln\left(\Phi\left(\frac{6 - \log_{10} L_{\text{model}}(t)}{\sigma_L}\right)\right) \tag{15}$$

where $\Phi(x)$ is the normal cumulative distribution function. For an observed relative PAD1 expression $P_{\text{data}}(t)$, at time t above the detection limit, the log-likelihood is proportional to

$$\ln\left(\phi\left(\frac{\log_2 P_{\text{data}}(t) - \log_2 P_{\text{model}}(t)}{\sigma_P}\right)\right) \tag{16}$$

The lowest $\log_2$ relative PAD1 expression is -7.52, we take this value as the detection limit, although it may be smaller. For a relative PAD1 expression below the detection limit at time t the log-likelihood is proportional to

$$\ln\left(\Phi\left(\frac{-7.52 - \log_2 P_{\text{model}}(t)}{\sigma_P}\right)\right) \tag{17}$$

The total log-likelihood for a mouse is the sum of the log-likelihoods over all the data for that mouse.

The parameter posterior distribution is found by multiplying the likelihood by the prior distributions, which are given in the next section. Samples from the posterior are drawn using an adaptive population based Markov chain Monte Carlo algorithm [7, 6].

2.2 Parameter Priors

The number of variant types ν that arose during the experiment is unknown. On the one hand there must be more than one because of the multiple peaks in parasite concentration, but on the other, we do not want the model to overfit the data with hundreds of variant types. We therefore chose a prior of $1 + \text{N}_T(0, 5)$ which allows for tens of variant types but penalises the model for having too many. (Note, $\text{N}_T(\mu, \sigma)$ is a normal probability density function with mean μ, standard deviation σ but truncated at 0.) Slender cell-cycle time has been estimated to be around 4-5 hours [9, 12], although [8] estimated it around 2 hours. We therefore chose a prior on α as $\text{N}_T(0.1, 0.1)$ in order to incorporate this uncertainty. [8] estimated the differentiation rate β to be between 0.5 and $3 \times 10^{-9}\text{h}^{-1}$, we therefore chose a prior of $\text{N}_T(10^{-9}, 10^{-9})$. SIF removal rate γ was estimated to be between 0.2 and 1.4h^{-1} [8], we therefore chose a prior $\text{N}_T(0.5, 0.5)$. There is no quantitative prior information about immune-mediated clearance of committed cells. It is thought, however, that the immune

system is less effective at clearing stumpy cells than slender cells [4, 2]. We therefore chose priors of U(0,1) for δ_c, δ_i and δ_s to reflect this uncertainty. [8] estimated the committed slender cell lifespan τ_c, to be between 8 and 12h, we therefore chose a prior of $\mathrm{N}_T(10, 4)$. [12] estimated the time from cell-cycle exit to the mitochondrion metabolic activity (when cells become morphologically stumpy) to be between 8 and 10h. [8] estimated this to be between 3 and 8h. We therefore chose a prior of $\mathrm{N}_T(6, 6)$ on the lifespan of intermediate cells τ_i. [1] estimated the lifespan of stumpy cells τ_s, to be between 24 and 36h. [10] estimated it to be between 48 and 72h, and [8] around 58h but with very wide confidence intervals. We therefore chose a prior of $\mathrm{N}_T(48, 24)$ to cover this uncertainty. The initial rise in PAD1 expression is thought to last until the end of the intermediate stage. Given the priors on τ_c and τ_i we set a prior on τ_p of $\mathrm{N}_T(10+6, \sqrt{4^2 + 6^2}) = \mathrm{N}_T(16, 7)$. Peak total relative PAD1 expression is about 2^5. This occurs at a peak parasitaemia of around $10^{8.5}$parasites$\,\mathrm{ml}^{-1}$ which is composed of mainly stumpy cells. Therefore relative PAD1 expression per stumpy cell is about $\frac{2^5}{10^{8.5}} = 10^{-7}$. Therefore we chose a prior of $\mathrm{N}_T(10^{-7}, 10^{-7})$ on ρ_1 and ρ_2. [8] suggested that committed slender cells produced SIF, we therefore set the prior for the duration of SIF production of committed cells τ_f as $\mathrm{N}_T(10, 4)$. Mice were infected intra-peritoneally with about 10^3 slender cells. We therefore have a prior of $N(3, 0.5)$ on $\log_{10} \lambda$. In [3] switch rates between variants were allowed to vary over six orders of magnitude with an average switch rate of 0.01 per population doubling, which is about 0.0014h^{-1} for a cell-cycle time of 7h. We therefore had a very broad prior on $\log_{10} \omega_v$ of N(-6,2). The prior on the initial activation of the immune response τ_r, was taken as $\mathrm{N}_T(168, 24)$, i.e., around 7 days as is usual in acute infectious diseases of naive animals. The growth of the immune responses against variants should occur on the order of several days. We therefore take broad priors on ϕ and ψ of $\mathrm{N}_T(0.1, 0.1)$ and $\mathrm{N}_T(10^{-8}, 10^{-8})$ respectively.

3 Results

3.1 Adequacy of fit

The fit of the model to parasite concentration and relative PAD1 expression to each mouse is shown in Fig. 4 in the main text. The dark and light grey regions are, respectively, the 50 and 95% posterior predictive intervals of the dynamics of these two variables. They represent our uncertainty in the dynamics of these variables given the data and the model. Although the fits appear good, a better assessment of adequacy of fit is to plot the standardised residuals; these are shown as crosses in Supp. Fig. 4a. Poor fits to data exhibit outliers (larger than about 3 standard deviations from 0). Systematic biases in fits across mice can be seen by plotting the mean standardised residual at each time point. The dashed lines represent the Bonferroni corrected 95% prediction interval of the mean were the true model [7]. Mean standardised residuals that lie above or below the interval suggest systematic under- and over-estimation of the data across mice.

3.2 SIF- or concentration-induced differentiation?

The standardised residuals of the model with concentration-induced differentiation are shown in Supp. Fig. 4b. This model gives a poor fit to the data with several outliers and systematic biases particularly around the first peak. The ratio of the two model's marginalised likelihoods tell us how much likely the data are under one model compared to the other. The marginalised likelihoods are products over all mice because we assume that the data from each mouse is independent of the data from all the other mice, i.e.,

$$\text{Prob}(\text{data} \mid \text{model}) = \prod_{i=1}^{6} \text{Prob}(\text{data from mouse } i \mid \text{model}) \tag{18}$$

The marginalised likelihoods of the two models are calculated by the model fitting algorithm: these are Prob(data | SIF model) = e^{-485} and Prob(data | Conc. model) = e^{-624}. Therefore the likelihood ratio is

$$\frac{\text{Prob}(\text{data} \mid \text{SIF model})}{\text{Prob}(\text{data} \mid \text{Conc. model})} = e^{139} \approx 10^{60} \tag{19}$$

Thus the data are overwhelmingly more likely under the SIF-induced differentiation model than the concentration-induced differentiation model.

3.3 Parameter estimates

Supplementary Figs. 5 and 6 display the marginalised parameter posterior distributions for each mouse. Some things to note

- Posterior distributions of intermediate cell lifespan contains 0. This means that this data alone cannot resolve this parameter.
- Committed cell-lifespan equals $\tau_c + \tau_i + \tau_s$.
- The number of committed slender cell replications equals $\frac{\tau_c \alpha}{\ln 2}$. They replicate between 2 to 4 times before becoming cell-cycle arrested.

3.4 Variant dynamics

The median predicted dynamics of the variant types are shown in Supp. Fig. 8. The first peak predominantly consists of one variant type. Notice that, even though switching between variants is sequential, it is still possible to get multiple variants arising simultaneously (mice 2 and 3, for example). For mouse 5 the rate of decline of the variant dynamics is much slower than in the other mice. This is because the immune-mediated clearance rates do not rise as high in this mouse compared to the other mice. This explains why the parasite concentration in this mouse remains high after the first peak (Fig. 4 in the main text).

References

[1] S. J. Black, R. S. Hewett, and C. N. Sendashonga. *Trypanosoma brucei* variable surface antigen is released by degenerating parasites but not by actively dividing parasites. *Parasite Immunology*, 4:233–244, 1982.

[2] M. Engstler, T. Pfohl, S. Herminghaus, M. Boshart, G. Wiegertjes, N. Heddergott, and P. Overath. Hydrodynamic flow-mediated protein sorting on the cell surface of trypanosomes. *Cell*, 131:505–515, 2007.

[3] K. A. Lythgoe, L. J. Morrison, A. F. Read, and D. Barry. Parasite-intrinsic factors can explain ordered progression of trypanosome antigenic variation. *PNAS*, 104(8095-8100), 2007.

[4] L. M. McLintock, C. M. Turner, and K. Vickerman. Comparison of the effects of immune killing mechanisms on *Trypanosoma brucei* parasites of slender and stumpy morphology. *Parasite Immunology*, 15:475–480, 1993.

[5] N. Mideo, V. C. Barclay, B. H. K. Chan, N. J. Savill, A. F. Read, and T. Day. Understanding and predicting strain-specific patterns of pathogenesis in the rodent malaria *Plasmodium chabaudi*. *American Naturalist*, 172(5):E214–E238, 2008.

[6] M. R. Miller, L. Råberg, A. F. Read, and N. J. Savill. Quantitative analysis of immune response and erythropoiesis during rodent malarial infection. *PloS Comp. Biol.*, 2010. in press.

[7] N. J. Savill, W. Chadwick, and S. E. Reece. Quantitative analysis of mechanisms that govern red blood cell age structure and dynamics during anaemia. *PLoS Computational Biology*, 5(6), 2009.

[8] N. J. Savill and J. R. Seed. Mathematical and statistical analysis of the *Trypanosoma brucei* slender to stumpy transition. *Parasitology*, 128(Part 1):53–67, 2004.

[9] J. R. Seed and S. J. Black. A proposed density–dependent model of long slender to short stumpy transformation in the African trypanosomes. *Journal of Parasitology*, 83:656–662, 1997.

[10] C. M. R. Turner, N. Aslam, and C. Dye. Replication, differentiation, growth and the virulence of *Trypanosoma brucei* infections. *Parasitology*, 111:289–300, 1995.

[11] K. M. Tyler, P. G. Higgs, K. R. Matthews, and K. Gull. Limitation of *Trypanosoma brucei* parasitemia results from density-dependent parasite differentiation and parasite killing by the host immune response. *Proceedings of the Royal Society London B*, 268:2235–2243, 2001.

[12] E. Vassella, B. Reuner, B. Yutzy, and M. Boshart. Differentiation of African trypanosomes is controlled by a density sensing mechanism which signals cell cycle arrest via the cAMP pathway. *Journal of Cell Science*, 110:2661–2671, 1997.
